# 1,3-Bis(3-phenyl­prop­yl)-1*H*-benzimidazol-3-ium-2-carbodithio­ate

**DOI:** 10.1107/S1600536808042761

**Published:** 2008-12-20

**Authors:** Mehmet Akkurt, Ülkü Yılmaz, Hasan Küçükbay, Mustafa Gençaslan, Orhan Büyükgüngör

**Affiliations:** a Department of Physics, Faculty of Arts and Sciences, Erciyes University, 38039 Kayseri, Turkey; b Department of Chemistry, Faculty of Arts and Sciences, nönü University, 44280 Malatya, Turkey; c Department of Physics, Faculty of Arts and Sciences, Ondokuz Mayıs University, 55139 Samsun, Turkey

## Abstract

The title compound, C_26_H_26_N_2_S_2_, was synthesized from bis­[1,3-bis­(3-phenyl­prop­yl)benzimidazolidine-2-yl­idene] and CS_2_ in toluene. The mol­ecular structure is composed of a benzimidazole ring system with two phenyl­propyl substituents and a dithio­carboxyl­ate group in the 2-position. The benzimidazole unit is essentially planar, with a maximum atomic deviation of 0.008 (2) Å, and makes dihedral angles of 72.72 (10) and 27.62 (12)°, with the two phenyl rings. The dihedral angle between the two phenyl rings is 55.98 (15)°. The mol­ecular packing is stabilized by a C—H⋯S inter­molecular hydrogen-bonding inter­action and a C—H⋯π inter­action between a benzene H atom and the phenyl ring of a neighbouring mol­ecule.

## Related literature

For applications of benzimidazole derivatives, see: Hahn & Jahnke (2008 ▶); Lappert (2005 ▶); Winberg & Coffman (1965 ▶); Küçükbay *et al.* (1996 ▶, 1997 ▶); Çetinkaya *et al.* (1994 ▶, 1998 ▶). For details of the synthesis, see: Yılmaz (2008 ▶). For related structures, see: Akkurt *et al.* (2004 ▶, 2005 ▶); Öztürk *et al.* (2003 ▶, 2004 ▶).
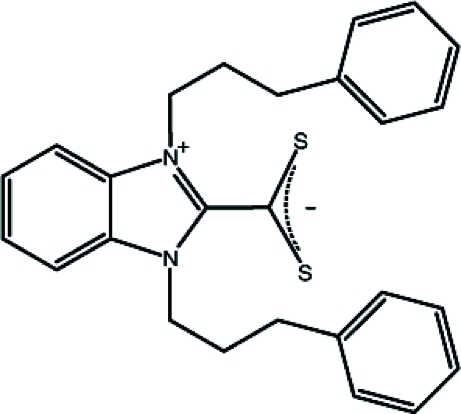


## Experimental

### 

#### Crystal data

C_26_H_26_N_2_S_2_
*M* *_r_* = 430.63
Orthorhombic, 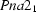

*a* = 27.2391 (11) Å
*b* = 8.3483 (4) Å
*c* = 10.3200 (4) Å
*V* = 2346.77 (17) Å^3^
*Z* = 4
Mo *K*α radiation
μ = 0.24 mm^−1^
*T* = 293 (2) K
0.76 × 0.65 × 0.38 mm

#### Data collection

Stoe IPDS-2 diffractometer
Absorption correction: integration (*X-RED32*; Stoe & Cie, 2002 ▶) *T*_min_ = 0.838, *T*_max_ = 0.914
19766 measured reflections
4983 independent reflections
3842 reflections with *I* > 2σ(*I*)
*R*_int_ = 0.029

#### Refinement

*R*[*F* ^2^ > 2σ(*F* ^2^)] = 0.032
*wR*(*F* ^2^) = 0.080
*S* = 0.97
4983 reflections
271 parameters
1 restraint
H-atom parameters constrained
Δρ_max_ = 0.10 e Å^−3^
Δρ_min_ = −0.19 e Å^−3^
Absolute structure: Flack (1983 ▶), 2317 Friedel pairs
Flack parameter: −0.03 (5)

### 

Data collection: *X-AREA* (Stoe & Cie, 2002 ▶); cell refinement: *X-AREA*; data reduction: *X-RED32* (Stoe & Cie, 2002 ▶); program(s) used to solve structure: *SIR97* (Altomare *et al.*, 1999 ▶); program(s) used to refine structure: *SHELXL97* (Sheldrick, 2008 ▶); molecular graphics: *ORTEP-3 for Windows* (Farrugia, 1997 ▶); software used to prepare material for publication: *WinGX* (Farrugia, 1999 ▶).

## Supplementary Material

Crystal structure: contains datablocks global, I. DOI: 10.1107/S1600536808042761/kj2110sup1.cif

Structure factors: contains datablocks I. DOI: 10.1107/S1600536808042761/kj2110Isup2.hkl

Additional supplementary materials:  crystallographic information; 3D view; checkCIF report

## Figures and Tables

**Table 1:** Selected bond lengths (Å)

C8—S1	1.6670 (18)
C8—S2	1.6532 (18)
C1—N1	1.393 (2)
C7—N2	1.341 (2)

**Table 2:** Hydrogen-bond geometry (Å, °)

*D*—H⋯*A*	*D*—H	H⋯*A*	*D*⋯*A*	*D*—H⋯*A*
C19—H19*B*⋯S2^i^	0.97	2.87	3.680 (2)	142
C5—H5⋯*Cg*1^ii^	0.93	2.70	3.523 (2)	148

Symmetry codes: (i) 
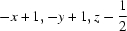
; (ii) 
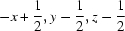
. *Cg*1 is the centroid of the C12–C17 phenyl ring.

**Fig. 1.**
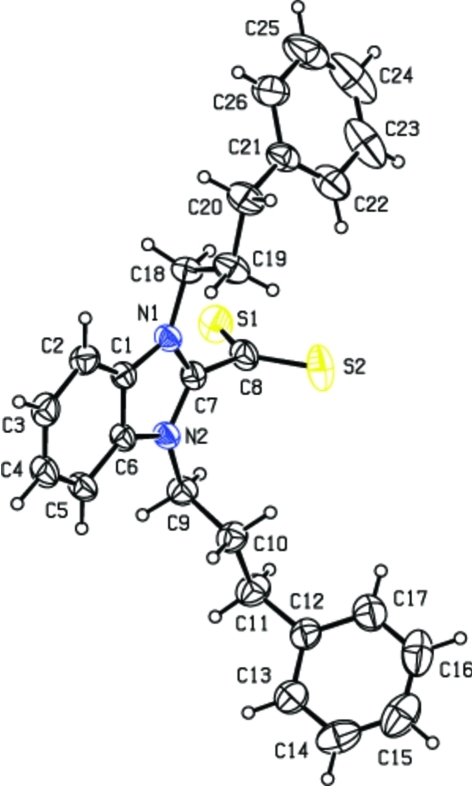
View of the title molecule, with the atom numbering scheme. Displacement ellipsoids for non-H atoms are drawn at the 30% probability level.

## supplementary crystallographic information

### Comment

Benzimidazole derivatives consistute an important class of heterocyclic compounds for their biological activities. They also are an important source for electron-rich olefin synthesis. Electron rich olefins are an important research subject for their versatile reactions. Since electron rich olefins are powerful nucleophilic compounds, they have been used as reducing agents, and are a source of carbine transation metal complexes and formylating agents for the proton active compounds (Hahn & Jahnke, 2008; Lappert, 2005). They are readily converted by carbon disulfide to red coloured stable dithioquaternary salts. (Winberg & Coffman,1965). Electron rich olefins have also been used as catalysts for cyloin type C—C coupling reactions. In a number of previous papers (Çetinkaya *et al.*, 1994; Küçükbay *et al.*, 1996; Küçükbay *et al.*, 1997; Çetinkaya, *et al.*, 1998) we reported the synthesis of some benzimidazole derived electron rich olefins.

The objective of this study was to elucidate the crystal structure of the title compound and to compare it with those of related benzimidazole derivatives reported previously (Akkurt *et al.*, 2004; Öztürk *et al.*, 2004; Akkurt *et al.*, 2005).

The *ORTEP* diagram of the title molecule with numbering scheme is shown in Fig. 1. The molecular structure of the title compound is composed of a benzimidazole ring with two phenylpropyl substituents and a dithiocarboxylate group in the 2-position. In the title molecule the C–S bonds are nearly equal in length. The N1–C7 and N2–C7 bond lengths in the benzimidazole ring agree well with several related benzimidazole derivatives (Öztürk *et al.*, 2003; Akkurt *et al.*, 2004). The benzimidazole unit (N1/N2/C1–C7) is essentially, with a maximum deviation from the least-squares plane of 0.008 (2) Å for C6. The benzimidazole ring makes dihedral angles of 72.72 (10) and 27.62 (12)°, with the two phenyl rings (C12–C17) and (C21–C26), respectively. The dihedral angle between the two phenyl rings is 55.98 (15)°.

The the molecular packing in the solid state is stabilized by a C–H···S type intermolecular hydrogen bonding interactions and a C—H···π interaction between a benzene H atom and the phenyl ring of neighbouring molecules, with a C5—H5···*Cg*1^ii^ separation of 2.70 Å [Table 2; *Cg*1 is the C12—C17 phenyl ring, symmetry code: (ii) -*x* + 1/2, *y* - 1/2, *z* - 1/2].

### Experimental

All experiments were performed under argon using freshly distilled dry solvents. CS_2 _(0.1 ml, 1.60 mmol) was added to a solution of bis[1,3-di(3-phenylpropyl)benzimidazolidine-2-ylidene] (0.55 g, 0.78 mmol) (Yılmaz, 2008) in toluene (5 ml). A red precipitate formed instantly. The red compound was washed twice with Et_2_O and crystallized from EtOH. [Yield: 0.60 g, 90%; m.p: 383–384 ° K]. Analysis calculated for C_26_H_26_N_2_S_2_: C 72.56, H 6.05, N 6.05, S 14.88%; found: C 71.99, H 5.93, N 6.30, S 14.18%.

### Refinement

H atoms were positioned geometrically and refined using a riding model, with C—H = 0.93 and C—H = 0.97 Å, and with *U*_iso_(H) = 1.2*U*_eq_(C).

### Crystal data

**Table:**

C_26_H_26_N_2_S_2_	*F*(000) = 912
*M**_r_* = 430.63	*D*_x_ = 1.219 Mg m^−^^3^
Orthorhombic, *P**n**a*2_1_	Mo *K*α radiation, λ = 0.71073 Å
Hall symbol: P 2c -2n	Cell parameters from 22533 reflections
*a* = 27.2391 (11) Å	θ = 1.5–27.3°
*b* = 8.3483 (4) Å	µ = 0.24 mm^−^^1^
*c* = 10.3200 (4) Å	*T* = 293 K
*V* = 2346.77 (17) Å^3^	Prism, red
*Z* = 4	0.76 × 0.65 × 0.38 mm

### Data collection

**Table:**

Stoe IPDS-2 diffractometer	4983 independent reflections
Radiation source: sealed X-ray tube, 12 x 0.4 mm long-fine focus	3842 reflections with *I* > 2σ(*I*)
plane graphite	*R*_int_ = 0.029
Detector resolution: 6.67 pixels mm^-1^	θ_max_ = 26.8°, θ_min_ = 1.5°
ω scans	*h* = −32→34
Absorption correction: integration (*X-RED32*; Stoe & Cie, 2002)	*k* = −10→10
*T*_min_ = 0.838, *T*_max_ = 0.914	*l* = −13→12
19766 measured reflections	

### Refinement

**Table:**

Refinement on *F*^2^	Secondary atom site location: difference Fourier map
Least-squares matrix: full	Hydrogen site location: inferred from neighbouring sites
*R*[*F*^2^ > 2σ(*F*^2^)] = 0.032	H-atom parameters constrained
*wR*(*F*^2^) = 0.080	*w* = 1/[σ^2^(*F*_o_^2^) + (0.0468*P*)^2^] where *P* = (*F*_o_^2^ + 2*F*_c_^2^)/3
*S* = 0.97	(Δ/σ)_max_ = 0.001
4983 reflections	Δρ_max_ = 0.10 e Å^−^^3^
271 parameters	Δρ_min_ = −0.19 e Å^−^^3^
1 restraint	Absolute structure: Flack (1983), 2317 Friedel pairs
Primary atom site location: structure-invariant direct methods	Flack parameter: −0.03 (5)

### Special details

**Table:**

Geometry. Bond distances, angles *etc*. have been calculated using the rounded fractional coordinates. All su's are estimated from the variances of the (full) variance-covariance matrix. The cell e.s.d.'s are taken into account in the estimation of distances, angles and torsion angles
Refinement. Refinement on *F*^2^ for ALL reflections except those flagged by the user for potential systematic errors. Weighted *R*-factors *wR* and all goodnesses of fit *S* are based on *F*^2^, conventional *R*-factors *R* are based on *F*, with *F* set to zero for negative *F*^2^. The observed criterion of *F*^2^ > σ(*F*^2^) is used only for calculating -*R*-factor-obs *etc*. and is not relevant to the choice of reflections for refinement. *R*-factors based on *F*^2^ are statistically about twice as large as those based on *F*, and *R*-factors based on ALL data will be even larger.

### Fractional atomic coordinates and isotropic or equivalent isotropic displacement parameters (Å^2^)

**Table:**

	*x*	*y*	*z*	*U*_iso_*/*U*_eq_	
S1	0.40082 (2)	0.05062 (6)	0.70350 (5)	0.0832 (2)	
S2	0.41878 (3)	0.40688 (8)	0.69551 (5)	0.0985 (2)	
N1	0.45770 (5)	0.20086 (16)	0.42158 (13)	0.0518 (4)	
N2	0.37896 (5)	0.24163 (16)	0.40602 (13)	0.0523 (4)	
C1	0.44694 (6)	0.2097 (2)	0.28979 (15)	0.0503 (5)	
C2	0.47649 (7)	0.1962 (2)	0.18080 (17)	0.0611 (6)	
C3	0.45312 (8)	0.2108 (2)	0.06250 (18)	0.0666 (7)	
C4	0.40275 (7)	0.2357 (2)	0.0538 (2)	0.0677 (7)	
C5	0.37380 (7)	0.2486 (2)	0.16044 (17)	0.0612 (6)	
C6	0.39687 (6)	0.2360 (2)	0.27995 (16)	0.0499 (5)	
C7	0.41636 (6)	0.22176 (19)	0.48903 (16)	0.0513 (5)	
C8	0.41218 (7)	0.2261 (2)	0.63249 (17)	0.0623 (6)	
C9	0.32764 (6)	0.2696 (2)	0.44304 (19)	0.0591 (6)	
C10	0.31416 (6)	0.4459 (2)	0.4397 (2)	0.0645 (6)	
C11	0.26262 (7)	0.4742 (2)	0.4912 (2)	0.0763 (8)	
C12	0.24982 (6)	0.6493 (2)	0.4969 (2)	0.0661 (6)	
C13	0.21865 (8)	0.7177 (3)	0.4061 (2)	0.0804 (8)	
C14	0.20761 (9)	0.8780 (4)	0.4092 (3)	0.1013 (11)	
C15	0.22701 (12)	0.9732 (3)	0.5006 (4)	0.1107 (13)	
C16	0.25833 (11)	0.9111 (4)	0.5923 (3)	0.1060 (11)	
C17	0.26948 (8)	0.7491 (3)	0.5899 (2)	0.0858 (9)	
C18	0.50755 (6)	0.1851 (2)	0.47476 (18)	0.0579 (6)	
C19	0.53581 (7)	0.3405 (2)	0.4620 (2)	0.0730 (7)	
C20	0.58916 (7)	0.3220 (3)	0.5025 (2)	0.0796 (8)	
C21	0.59723 (7)	0.2812 (2)	0.6424 (2)	0.0680 (7)	
C22	0.56779 (9)	0.3408 (3)	0.7394 (2)	0.0944 (10)	
C23	0.57637 (14)	0.3030 (5)	0.8670 (3)	0.1282 (14)	
C24	0.6143 (2)	0.2054 (6)	0.9002 (4)	0.1497 (19)	
C25	0.64464 (17)	0.1496 (3)	0.8060 (5)	0.1379 (18)	
C26	0.63577 (9)	0.1846 (3)	0.6778 (3)	0.0989 (10)	
H2	0.51010	0.17840	0.18700	0.0730*	
H3	0.47150	0.20370	−0.01320	0.0800*	
H4	0.38840	0.24380	−0.02770	0.0810*	
H5	0.34010	0.26510	0.15370	0.0740*	
H9A	0.32220	0.22840	0.52980	0.0710*	
H9B	0.30630	0.21130	0.38440	0.0710*	
H10A	0.31620	0.48470	0.35130	0.0770*	
H10B	0.33750	0.50590	0.49160	0.0770*	
H11A	0.23920	0.41940	0.43590	0.0920*	
H11B	0.26000	0.42870	0.57750	0.0920*	
H13	0.20500	0.65360	0.34180	0.0970*	
H14	0.18650	0.92100	0.34740	0.1210*	
H15	0.21930	1.08170	0.50190	0.1330*	
H16	0.27190	0.97710	0.65540	0.1270*	
H17	0.29060	0.70700	0.65200	0.1030*	
H18A	0.52490	0.10080	0.42900	0.0690*	
H18B	0.50560	0.15530	0.56540	0.0690*	
H19A	0.52040	0.42170	0.51550	0.0880*	
H19B	0.53450	0.37660	0.37270	0.0880*	
H20A	0.60400	0.23880	0.44980	0.0960*	
H20B	0.60620	0.42130	0.48370	0.0960*	
H22	0.54170	0.40770	0.71830	0.1130*	
H23	0.55610	0.34460	0.93120	0.1540*	
H24	0.61940	0.17720	0.98630	0.1800*	
H25	0.67160	0.08720	0.82830	0.1660*	
H26	0.65620	0.14220	0.61420	0.1190*	

### Atomic displacement parameters (Å^2^)

**Table:**

	*U*^11^	*U*^22^	*U*^33^	*U*^12^	*U*^13^	*U*^23^
S1	0.1109 (4)	0.0873 (3)	0.0513 (2)	0.0024 (3)	0.0091 (3)	0.0103 (3)
S2	0.1433 (5)	0.0944 (4)	0.0579 (3)	−0.0250 (4)	0.0058 (4)	−0.0251 (3)
N1	0.0519 (8)	0.0605 (8)	0.0429 (7)	0.0010 (6)	−0.0031 (6)	0.0001 (6)
N2	0.0503 (8)	0.0625 (8)	0.0441 (7)	0.0012 (6)	−0.0022 (6)	−0.0033 (6)
C1	0.0572 (10)	0.0544 (9)	0.0394 (8)	0.0040 (7)	−0.0024 (7)	−0.0024 (7)
C2	0.0618 (10)	0.0694 (10)	0.0520 (10)	0.0080 (8)	0.0083 (8)	0.0004 (8)
C3	0.0858 (14)	0.0719 (12)	0.0420 (9)	0.0060 (10)	0.0095 (9)	−0.0037 (8)
C4	0.0844 (14)	0.0778 (13)	0.0410 (9)	0.0022 (10)	−0.0081 (9)	−0.0028 (8)
C5	0.0624 (10)	0.0742 (11)	0.0471 (9)	−0.0003 (8)	−0.0106 (8)	−0.0002 (8)
C6	0.0559 (9)	0.0570 (9)	0.0369 (8)	−0.0006 (7)	−0.0020 (7)	−0.0018 (6)
C7	0.0575 (9)	0.0518 (8)	0.0446 (8)	−0.0019 (7)	−0.0018 (8)	−0.0034 (7)
C8	0.0633 (11)	0.0818 (12)	0.0418 (10)	−0.0001 (9)	−0.0001 (8)	−0.0062 (8)
C9	0.0479 (9)	0.0686 (11)	0.0608 (11)	−0.0019 (7)	0.0050 (8)	0.0002 (8)
C10	0.0541 (9)	0.0686 (11)	0.0709 (11)	0.0011 (8)	0.0114 (9)	−0.0043 (8)
C11	0.0579 (11)	0.0797 (13)	0.0914 (15)	0.0039 (9)	0.0140 (10)	−0.0032 (11)
C12	0.0496 (9)	0.0788 (12)	0.0699 (11)	0.0015 (8)	0.0152 (9)	−0.0109 (10)
C13	0.0646 (12)	0.1008 (16)	0.0759 (14)	0.0096 (11)	0.0135 (10)	−0.0116 (12)
C14	0.0818 (16)	0.110 (2)	0.112 (2)	0.0257 (15)	0.0257 (15)	0.0243 (17)
C15	0.0982 (19)	0.0830 (17)	0.151 (3)	−0.0029 (15)	0.051 (2)	−0.0083 (19)
C16	0.0969 (19)	0.107 (2)	0.114 (2)	−0.0291 (16)	0.0341 (17)	−0.0437 (17)
C17	0.0717 (13)	0.1021 (19)	0.0835 (16)	−0.0096 (12)	0.0062 (12)	−0.0137 (12)
C18	0.0524 (9)	0.0674 (10)	0.0539 (10)	0.0028 (7)	−0.0071 (8)	0.0086 (8)
C19	0.0684 (11)	0.0826 (13)	0.0679 (12)	−0.0119 (10)	−0.0139 (9)	0.0199 (10)
C20	0.0599 (12)	0.1052 (16)	0.0738 (13)	−0.0160 (10)	−0.0057 (10)	0.0108 (12)
C21	0.0585 (11)	0.0676 (11)	0.0779 (13)	−0.0108 (9)	−0.0156 (10)	0.0089 (9)
C22	0.0761 (15)	0.130 (2)	0.0772 (15)	−0.0115 (14)	−0.0137 (12)	−0.0058 (14)
C23	0.118 (2)	0.192 (3)	0.0747 (19)	−0.071 (2)	−0.0107 (18)	−0.006 (2)
C24	0.195 (4)	0.142 (3)	0.112 (3)	−0.098 (3)	−0.080 (3)	0.058 (2)
C25	0.163 (3)	0.0748 (17)	0.176 (4)	−0.0163 (19)	−0.104 (3)	0.037 (2)
C26	0.0874 (16)	0.0754 (13)	0.134 (2)	0.0046 (11)	−0.0398 (16)	−0.0007 (15)

### Geometric parameters (Å, °)

**Table:**

S1—C8	1.6670 (18)	C23—C24	1.360 (7)
S2—C8	1.6532 (18)	C24—C25	1.358 (7)
N1—C1	1.393 (2)	C25—C26	1.376 (6)
N1—C7	1.335 (2)	C2—H2	0.9300
N1—C18	1.471 (2)	C3—H3	0.9300
N2—C6	1.390 (2)	C4—H4	0.9300
N2—C7	1.341 (2)	C5—H5	0.9300
N2—C9	1.468 (2)	C9—H9A	0.9700
C1—C2	1.388 (2)	C9—H9B	0.9700
C1—C6	1.385 (2)	C10—H10A	0.9700
C2—C3	1.382 (3)	C10—H10B	0.9700
C3—C4	1.391 (3)	C11—H11A	0.9700
C4—C5	1.358 (3)	C11—H11B	0.9700
C5—C6	1.388 (2)	C13—H13	0.9300
C7—C8	1.485 (2)	C14—H14	0.9300
C9—C10	1.517 (2)	C15—H15	0.9300
C10—C11	1.520 (3)	C16—H16	0.9300
C11—C12	1.504 (2)	C17—H17	0.9300
C12—C13	1.388 (3)	C18—H18A	0.9700
C12—C17	1.379 (3)	C18—H18B	0.9700
C13—C14	1.372 (4)	C19—H19A	0.9700
C14—C15	1.342 (5)	C19—H19B	0.9700
C15—C16	1.376 (5)	C20—H20A	0.9700
C16—C17	1.386 (4)	C20—H20B	0.9700
C18—C19	1.514 (2)	C22—H22	0.9300
C19—C20	1.520 (3)	C23—H23	0.9300
C20—C21	1.500 (3)	C24—H24	0.9300
C21—C22	1.376 (3)	C25—H25	0.9300
C21—C26	1.373 (3)	C26—H26	0.9300
C22—C23	1.374 (4)		
			
S1···N1	3.5268 (15)	C21···H18B	2.8200
S1···N2	3.5103 (14)	C22···H18B	2.9100
S2···N1	3.4747 (15)	C22···H19A	2.7300
S2···N2	3.4648 (15)	C24···H10B^i^	2.9000
S2···C19^i^	3.680 (2)	H2···C18	2.9700
S1···H24^ii^	2.9900	H2···S1^ii^	3.0900
S1···H2^iii^	3.0900	H4···C13^iv^	3.0000
S1···H9A	3.1600	H4···C14^iv^	2.9200
S2···H10B	3.1600	H5···C9	3.0100
S2···H19B^i^	2.8700	H5···H9B	2.5900
N1···S1	3.5268 (15)	H5···C12^iv^	3.0900
N1···S2	3.4747 (15)	H5···C13^iv^	3.0400
N1···N2	2.1776 (19)	H5···C14^iv^	2.9900
N2···S1	3.5103 (14)	H5···C15^iv^	2.9800
N2···S2	3.4648 (15)	H5···C16^iv^	3.0100
N2···N1	2.1776 (19)	H5···C17^iv^	3.0600
C2···C19	3.533 (3)	H9A···S1	3.1600
C3···C18^ii^	3.590 (2)	H9A···C8	2.6700
C4···C14^iv^	3.560 (3)	H9A···H11B	2.4300
C5···C14^iv^	3.579 (3)	H9B···C5	2.9700
C6···C25^ii^	3.423 (3)	H9B···H5	2.5900
C10···C24^v^	3.527 (5)	H9B···H11A	2.5800
C14···C5^vi^	3.579 (3)	H10B···S2	3.1600
C14···C4^vi^	3.560 (3)	H10B···C17	2.9300
C18···C3^iii^	3.590 (2)	H10B···C24^v^	2.9000
C18···C22	3.441 (3)	H11A···H9B	2.5800
C19···C2	3.533 (3)	H11A···H13	2.3700
C19···S2^v^	3.680 (2)	H11B···H9A	2.4300
C22···C18	3.441 (3)	H11B···H17	2.5900
C24···C10^i^	3.527 (5)	H13···H11A	2.3700
C25···C6^iii^	3.423 (3)	H13···H16^iv^	2.5000
C1···H19B	2.8900	H16···H13^vi^	2.5000
C2···H18A	2.9900	H17···H11B	2.5900
C2···H19B	2.9500	H18A···C2	2.9900
C3···H18A^ii^	3.0000	H18A···H20A	2.4500
C4···H20B^v^	2.9600	H18A···C3^iii^	3.0000
C5···H9B	2.9700	H18B···C8	2.7000
C8···H18B	2.7000	H18B···C21	2.8200
C8···H9A	2.6700	H18B···C22	2.9100
C9···H5	3.0100	H19A···C22	2.7300
C12···H5^vi^	3.0900	H19A···H22	2.1700
C13···H5^vi^	3.0400	H19B···C1	2.8900
C13···H4^vi^	3.0000	H19B···C2	2.9500
C14···H4^vi^	2.9200	H19B···S2^v^	2.8700
C14···H5^vi^	2.9900	H20A···H18A	2.4500
C15···H5^vi^	2.9800	H20A···H26	2.3600
C16···H5^vi^	3.0100	H20B···C4^i^	2.9600
C17···H5^vi^	3.0600	H22···C19	2.7100
C17···H10B	2.9300	H22···H19A	2.1700
C18···H2	2.9700	H24···S1^iii^	2.9900
C19···H22	2.7100	H26···H20A	2.3600
			
C1—N1—C7	108.94 (13)	C6—C5—H5	122.00
C1—N1—C18	124.29 (14)	N2—C9—H9A	109.00
C7—N1—C18	126.57 (14)	N2—C9—H9B	109.00
C6—N2—C7	109.09 (13)	C10—C9—H9A	109.00
C6—N2—C9	125.67 (14)	C10—C9—H9B	109.00
C7—N2—C9	125.22 (14)	H9A—C9—H9B	108.00
N1—C1—C2	131.68 (16)	C9—C10—H10A	109.00
N1—C1—C6	106.68 (14)	C9—C10—H10B	109.00
C2—C1—C6	121.64 (15)	C11—C10—H10A	109.00
C1—C2—C3	116.21 (17)	C11—C10—H10B	109.00
C2—C3—C4	121.64 (18)	H10A—C10—H10B	108.00
C3—C4—C5	122.17 (18)	C10—C11—H11A	109.00
C4—C5—C6	116.81 (17)	C10—C11—H11B	109.00
N2—C6—C1	106.40 (14)	C12—C11—H11A	109.00
N2—C6—C5	132.07 (16)	C12—C11—H11B	109.00
C1—C6—C5	121.53 (16)	H11A—C11—H11B	108.00
N1—C7—N2	108.89 (14)	C12—C13—H13	119.00
N1—C7—C8	125.98 (15)	C14—C13—H13	119.00
N2—C7—C8	125.12 (15)	C13—C14—H14	120.00
S1—C8—S2	130.51 (11)	C15—C14—H14	120.00
S1—C8—C7	115.53 (12)	C14—C15—H15	120.00
S2—C8—C7	113.96 (12)	C16—C15—H15	120.00
N2—C9—C10	112.26 (13)	C15—C16—H16	120.00
C9—C10—C11	111.50 (14)	C17—C16—H16	120.00
C10—C11—C12	112.27 (14)	C12—C17—H17	119.00
C11—C12—C13	121.03 (18)	C16—C17—H17	119.00
C11—C12—C17	121.63 (18)	N1—C18—H18A	109.00
C13—C12—C17	117.32 (18)	N1—C18—H18B	109.00
C12—C13—C14	121.3 (2)	C19—C18—H18A	109.00
C13—C14—C15	120.5 (3)	C19—C18—H18B	109.00
C14—C15—C16	120.3 (3)	H18A—C18—H18B	108.00
C15—C16—C17	119.4 (3)	C18—C19—H19A	109.00
C12—C17—C16	121.1 (2)	C18—C19—H19B	109.00
N1—C18—C19	111.12 (14)	C20—C19—H19A	109.00
C18—C19—C20	112.03 (16)	C20—C19—H19B	109.00
C19—C20—C21	115.34 (17)	H19A—C19—H19B	108.00
C20—C21—C22	122.21 (18)	C19—C20—H20A	108.00
C20—C21—C26	120.1 (2)	C19—C20—H20B	108.00
C22—C21—C26	117.7 (2)	C21—C20—H20A	108.00
C21—C22—C23	121.0 (3)	C21—C20—H20B	108.00
C22—C23—C24	120.6 (3)	H20A—C20—H20B	107.00
C23—C24—C25	119.2 (4)	C21—C22—H22	119.00
C24—C25—C26	120.6 (4)	C23—C22—H22	119.00
C21—C26—C25	121.0 (3)	C22—C23—H23	120.00
C1—C2—H2	122.00	C24—C23—H23	120.00
C3—C2—H2	122.00	C23—C24—H24	120.00
C2—C3—H3	119.00	C25—C24—H24	120.00
C4—C3—H3	119.00	C24—C25—H25	120.00
C3—C4—H4	119.00	C26—C25—H25	120.00
C5—C4—H4	119.00	C21—C26—H26	120.00
C4—C5—H5	122.00	C25—C26—H26	119.00
			
C7—N1—C1—C2	179.97 (18)	C4—C5—C6—C1	−0.7 (2)
C18—N1—C1—C2	4.8 (3)	N1—C7—C8—S2	89.55 (19)
C7—N1—C1—C6	−0.39 (18)	N2—C7—C8—S1	90.04 (19)
C18—N1—C1—C6	−175.53 (14)	N1—C7—C8—S1	−91.39 (19)
C1—N1—C7—N2	0.86 (18)	N2—C7—C8—S2	−89.03 (19)
C18—N1—C7—N2	175.86 (14)	N2—C9—C10—C11	−174.26 (15)
C1—N1—C7—C8	−177.91 (15)	C9—C10—C11—C12	176.22 (16)
C18—N1—C7—C8	−2.9 (3)	C10—C11—C12—C13	104.4 (2)
C7—N1—C18—C19	−101.35 (19)	C10—C11—C12—C17	−73.8 (2)
C1—N1—C18—C19	72.9 (2)	C11—C12—C13—C14	−178.7 (2)
C9—N2—C7—N1	−179.33 (14)	C13—C12—C17—C16	0.2 (3)
C6—N2—C7—C8	177.78 (15)	C17—C12—C13—C14	−0.4 (3)
C6—N2—C7—N1	−1.00 (18)	C11—C12—C17—C16	178.4 (2)
C9—N2—C6—C1	179.06 (15)	C12—C13—C14—C15	0.3 (4)
C9—N2—C7—C8	−0.6 (2)	C13—C14—C15—C16	0.1 (5)
C9—N2—C6—C5	−2.3 (3)	C14—C15—C16—C17	−0.3 (5)
C7—N2—C9—C10	97.6 (2)	C15—C16—C17—C12	0.2 (4)
C7—N2—C6—C5	179.42 (18)	N1—C18—C19—C20	−173.65 (15)
C6—N2—C9—C10	−80.5 (2)	C18—C19—C20—C21	−64.2 (2)
C7—N2—C6—C1	0.74 (18)	C19—C20—C21—C22	−35.6 (3)
C6—C1—C2—C3	0.1 (2)	C19—C20—C21—C26	146.1 (2)
N1—C1—C2—C3	179.71 (17)	C20—C21—C22—C23	−179.2 (3)
N1—C1—C6—C5	−179.06 (15)	C26—C21—C22—C23	−0.9 (4)
C2—C1—C6—N2	179.47 (15)	C20—C21—C26—C25	178.1 (2)
N1—C1—C6—N2	−0.21 (18)	C22—C21—C26—C25	−0.3 (4)
C2—C1—C6—C5	0.6 (3)	C21—C22—C23—C24	0.0 (5)
C1—C2—C3—C4	−0.7 (2)	C22—C23—C24—C25	2.1 (7)
C2—C3—C4—C5	0.6 (3)	C23—C24—C25—C26	−3.2 (6)
C3—C4—C5—C6	0.1 (3)	C24—C25—C26—C21	2.4 (5)
C4—C5—C6—N2	−179.24 (17)		

Symmetry codes: (i) −*x*+1, −*y*+1, *z*+1/2; (ii) −*x*+1, −*y*, *z*−1/2; (iii) −*x*+1, −*y*, *z*+1/2; (iv) −*x*+1/2, *y*−1/2, *z*−1/2; (v) −*x*+1, −*y*+1, *z*−1/2; (vi) −*x*+1/2, *y*+1/2, *z*+1/2.

### Hydrogen-bond geometry (Å, °)

**Table:**

*D*—H···*A*	*D*—H	H···*A*	*D*···*A*	*D*—H···*A*
C19—H19B···S2^v^	0.97	2.87	3.680 (2)	142
C5—H5···Cg1^iv^	0.93	2.70	3.523 (2)	148

Symmetry codes: (v) −*x*+1, −*y*+1, *z*−1/2; (iv) −*x*+1/2, *y*−1/2, *z*−1/2.

## References

[bb1] Akkurt, M., Karaca, S., Küçükbay, H., Yılmaz, U. & Büyükgüngör, O. (2005). *Acta Cryst.* E**61**, o2875–o2877.

[bb2] Akkurt, M., Öztürk, S., Küçükbay, H., Orhan, E. & Büyükgüngör, O. (2004). *Acta Cryst.* E**60**, o219–o221.

[bb3] Altomare, A., Burla, M. C., Camalli, M., Cascarano, G. L., Giacovazzo, C., Guagliardi, A., Moliterni, A. G. G., Polidori, G. & Spagna, R. (1999). *J. Appl. Cryst.* **32**, 115–119.

[bb4] Çetinkaya, B., Çetinkaya, E., Chamizo, J. A., Hitchcock, P. B., Jasim, H. A., Küçükbay, H. & Lappert, M. F. (1998). *J. Chem. Soc. Perkin Trans. 1*, pp. 2047–2054.

[bb5] Çetinkaya, E., Hitchcock, P. B., Küçükbay, H. & Lappert, M. F. (1994). *J. Organomet. Chem.* **481**, 89–95.

[bb6] Farrugia, L. J. (1997). *J. Appl. Cryst.* **30**, 565.

[bb7] Farrugia, L. J. (1999). *J. Appl. Cryst.* **32**, 837–838.

[bb8] Flack, H. D. (1983). *Acta Cryst.* A**39**, 876–881.

[bb9] Hahn, F. E. & Jahnke, M. (2008). *Angew. Chem. Int. Ed.* **47**, 3122–3172. 10.1002/anie.200703883 18398856

[bb10] Küçükbay, H., Çetinkaya, E., Çetinkaya, B. & Lappert, M. F. (1997). *Synth. Commun.* **27**, 4059–4066.

[bb11] Küçükbay, H., Çetinkaya, B., Guesmi, S. & Dixneuf, P. H. (1996). *Organometallics*, **15**, 2434–2439.

[bb12] Lappert, M. F. (2005). *J. Organomet. Chem.* **690**, 5467–5473.

[bb13] Öztürk, S., Akkurt, M., Küçükbay, H., Okuyucu, N. & Fun, H.-K. (2003). *Acta Cryst.* E**59**, o1014–o1016.

[bb14] Öztürk, S., Akkurt, M., Küçükbay, H., Orhan, E. & Büyükgüngör, O. (2004). *Acta Cryst.* E**60**, o936–o938.

[bb15] Sheldrick, G. M. (2008). *Acta Cryst.* A**64**, 112–122. 10.1107/S0108767307043930 18156677

[bb16] Stoe & Cie (2002). *X-AREA*and *X-RED32* Stoe & Cie, Darmstadt, Germany.

[bb17] Winberg, H. E. & Coffman, D. D. (1965). *J. Am. Chem. Soc.* **87**, 2776–2777.

[bb18] Yılmaz, Ü. (2008). PhD thesis, İnönü University, Graduate School of Natural and Applied Sciences, Malatya, Turkey.

